# Metabolic engineering of *Mortierella alpina* for arachidonic acid production with glycerol as carbon source

**DOI:** 10.1186/s12934-015-0392-4

**Published:** 2015-12-23

**Authors:** Guangfei Hao, Haiqin Chen, Zhennan Gu, Hao Zhang, Wei Chen, Yong Q. Chen

**Affiliations:** State Key Laboratory of Food Science and Technology, School of Food Science and Technology, Jiangnan University, Wuxi, 214122 People’s Republic of China; Synergistic Innovation Center for Food Safety and Nutrition, Wuxi, 214122 People’s Republic of China; Beijing Innovation Centre of Food Nutrition and Human Health, Beijing Technology and Business University (BTBU), Beijing, 100048 People’s Republic of China; Departments of Cancer Biology and Biochemistry, Wake Forest School of Medicine, Winston-Salem, NC 27157 USA

**Keywords:** *Mortierella alpina*, Fatty acid production, Raw glycerol, NADPH

## Abstract

**Background:**

Although some microorganisms can convert glycerol into valuable products such as polyunsaturated fatty acids, the yields are relative low due primarily to an inefficient assimilation of glycerol. *Mortierella alpina* is an oleaginous fungus which preferentially uses glucose over glycerol as the carbon source for fatty acid synthesis.

**Results:**

In the present study, we metabolically engineered *M. alpina* to increase the utilization of glycerol. Glycerol kinase and glycerol-3-phosphate dehydrogenase control the first two steps of glycerol decomposition. GK overexpression increased the total fatty acid content by 35 %, whereas G3PD1, G3PD2 and G3PD3 had no significant effect. Overexpression of malic enzyme (ME1) but not glucose-6-phosphate dehydrogenase, 6-phosphogluconate dehydrogenase or isocitrate dehydrogenase significantly increased fatty acid content when glycerol was used as carbon source. Simultaneous overexpression of GK and ME1 enabled *M. alpina* to accumulate fatty acids efficiently, with a 44 % increase in fatty acid content (% of dry weight), a 57 % increase in glycerol to fatty acid yield (g/g glycerol) and an 81 % increase in fatty acid production (g/L culture). A repeated batch process was applied to relieve the inhibitory effect of raw glycerol on arachidonic acid synthesis, and under these conditions, the yield reached 52.2 ± 1.9 mg/g.

**Conclusions:**

This study suggested that GK is a rate-limiting step in glycerol assimilation in *M. alpina*. Another restricting factor for fatty acid accumulation was the supply of cytosolic NADPH. We reported a bioengineering strategy by improving the upstream assimilation and NADPH supply, for oleaginous fungi to efficiently accumulate fatty acid with glycerol as carbon source.

**Electronic supplementary material:**

The online version of this article (doi:10.1186/s12934-015-0392-4) contains supplementary material, which is available to authorized users.

## Background

Biodiesel is a widely accepted renewable energy source that has been added to fossil fuels for many years [1]. Glycerol is the major biodiesel byproduct, which constitute approximately 80 % of the biodiesel-derived waste, and has become an environmental burden since it can neither be largely consumed by the traditional fermentation industry nor efficiently burned as fuel [2, 3]. Microbiological conversion of glycerol into organic chemical materials is a promising and rapidly developing solution [3–5]. In pursuit of a higher commercial added-value to relieve the pressure for cost-efficiency, the use of glycerol to produce polyunsaturated fatty acids (PUFAs) has gathered increasing interest in recent years [6].

Some oleaginous microorganisms can grow with glycerol as the sole carbon source to accumulate PUFAs [7–10]. When using glycerol as carbon source, the key issue is the relatively low assimilation efficiency that limits downstream metabolic processes. This is presumably due to the insufficient coordination of the enzymes involved in the primary metabolic steps of glycerol assimilation [7, 10, 11]. During aerobic growth, glycerol is catabolized by glycerol kinase (GK, EC 2.7.1.30) to glycerol-3-phosphate, which can be used either as a precursor for lipid biosynthesis or converted by glycerol-3-phosphate dehydrogenase (G3PD, EC 1.1.1.8) to dihydroxyacetone phosphate (DHAP) to enter the glycolysis pathway [12]. Previously, GK and G3PD have been demonstrated as the enzymes responsible for glycerol assimilation in the production of various compounds, including 1,2-propanediol [13], succinate [14], lactic acid [15], shikimic acid [16]. In DHA-rich microalgae *Schizochytrium*, GK and G3PD were also suggested to play a dominant role in glycerol assimilation [17]. Thus, overexpression of the genes encoding GK and G3PD is a promising way to improve glycerol assimilation for fatty acid production in oleaginous microbes.

In oleaginous microorganisms, NADPH is critical for fatty acid synthesis and is primarily generated from the pentose phosphate pathway (PPP) [18, 19]. In the absence of glucose, the PPP may be affected in several ways to cause a shortage of NADPH for fatty acid accumulation. This insufficiency can hardly be compensated by malic enzyme (ME, EC 1.1.1.40), because it is down-regulated at the transcriptional level during the fatty acid accumulation stage [20]. Isocitrate dehydrogenase (ICDH, EC 1.1.1.42) is believed to be another important NADPH source, but its role during fatty acid synthesis needs to be further characterized [21]. This may be another possible reason for the insufficient accumulation of fatty acids when organisms are cultured with glycerol as sole carbon source. For instance, NADPH also plays an important role in preventing cell damage caused by reactive oxygen species such as free radicals, peroxides, lipid peroxides and heavy metals [22, 23]. In cultures with raw glycerol, the insufficient NADPH generation may exacerbate the inhibitory effect of residual components such as soap, sodium or potassium salts, methanol and heavy metals on cell proliferation and metabolism [24, 25]. As the purification process is uneconomical for the downstream industrial utilization [3, 24], directly using raw glycerol as feedstock for fermentation will remain the most reasonable choice for future applications. Thus, improvement of the NADPH supplementation is required for oleaginous microbes to accumulate fatty acids when cultured with raw glycerol.

**Fig. 1:**
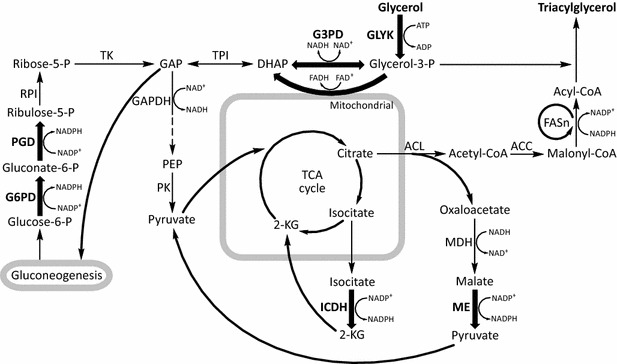
Overview of the metabolic pathways for fatty acid synthesis with glycerol as carbon source in *M. alpina*. *DHAP* dihydroxyacetone phosphate; *GAP* glyceraldehyde 3-phosphate; *PEP* phosphoenolpyruvate; *2-KG* 2-ketoglutarate; *GK* glycerol kinase; *G3PD* glycerol-3-phosphate dehydrogenase; *TPI* triose phosphate isomerase; *GAPDH* glyceraldehyde-3-phosphate dehydrogenase; *PK* pyruvate kinase; *G6PD* Glucose-6-phosphate dehydrogenase; *PGD* 6-phosphogluconate dehydrogenase; *RPI* ribose-5-phosphate isomerase; *TK* transketolase; *ICDH* isocitrate dehydrogenase; *ACL ATP* citrate synthase; *MDH* malate dehydrogenase; *ME* malic enzyme; *ACC* acetyl-CoA carboxylase; *FASn* fatty acid synthase

In this study, industrial oleaginous fungus *Mortierella alpina* was used to study arachidonic acid (ARA) production with glycerol as sole carbon source. *M. alpina* has been previously reported to be able to assimilate glycerol and accumulate ARA, but the biomass and ARA production were significantly affected [25, 26]. The present work aimed at improving PUFAs yield by genetically modifying the initial assimilation steps and the availability of NADPH in *M. alpina* cultured with glycerol (Fig. 1).

## Results

### GK and G3PD expression levels during lipogenesis in *M. alpina* with different carbon sources

**Fig. 2:**
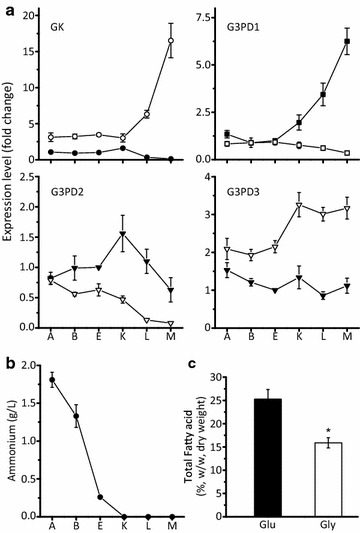
**a** Expression levels of GK and G3PDs in *M. alpina* growing with glucose or glycerol as carbon source. *M. alpina* was cultured in a 7.5 L fermenter and sampled at various time points prior to and after nitrogen exhaustion (sample A: −12 h, B: −2 h, E: −30 min, K: +1 h, L: +12 h and M: +48 h), and transcript levels were analyzed by RT-qPCR. Filled symbols represent the fold change of expression of GK and G3PDs prior to and after the onset of lipogenesis (time point E) in *M. alpina* growing with glucose. Empty *symbols* represent the fold change in transcript levels of GK and G3PDs in *M. alpina* growing with glycerol compared to that growing with glucose at the same time points. **b** Fatty acid content of *M. alpina* cultured with glucose or glycerol as sole carbon source. *M. alpina* was cultured in a 500 mL shaking flask containing Kendrick medium with glucose (*filled bar*) or glycerol (*empty bar*) for 168 h. Three independent experiments were performed, and the error bars represent standard deviations. *p < 0.05 compared to wild type

First, we analyzed the expression level of GK and G3PDs in a series *M. alpina* samples prior to (sample A: −12 h, B: −2 h, E: −30 min) and after (sample K: +1 h, L: +12 h and M: +48 h) nitrogen exhaustion during fatty acid synthesis by RT-qPCR as previously described [18]. When samples were cultured with glucose, the expression of GK kept decreasing to reach an extremely low level during the fatty acid accumulation stage (K, L, M). Meanwhile, the expression of G3PD1 (NAD^+^) was increased more than fivefold, and the expression of the other G3PDs was not significantly downregulated (Fig. 2a) after nitrogen exhaustion (Fig. 2b). These results were consistent with the previously performed transcriptome analysis, and indicated that the G3PDs may play an important role during fatty acid accumulation in *M. alpina* [18]. When samples were cultured with glycerol, the expression level of GK was significantly increased by more than 15-fold to enable the first assimilation step of glycerol. By contrast, the G3PDs were differently regulated: NAD^+^ dependent G3PD1 and G3PD2 were significantly downregulated at the transcript level, while the expression of FAD^+^ dependent G3PD3 increased by more than twofold (Fig. 2a). This may indicate that GK and G3PD3 play important roles during glycerol assimilation. G3PD1 and G3PD2 were still moderately expressed, so they may also be functional.

### Overexpression of GK and G3PDs in *M. alpina*

When *M. alpina* was cultured with glycerol, the fatty acid content and PUFAs amounts were significantly lower than in the presence of glucose (Fig. 2c, Table 1), indicating a lower fatty acid accumulation and desaturation efficiency. However, no negative impact of glycerol on the content of ARA was observed (Table 1), which is consistent with previously reported data [27]. Due to the decrease of the biomass and fatty acids (from 10.3 ± 0.6 to 8.1 ± 0.3 g/L and from 2.6 ± 0.2 to 1.3 ± 0.1 g/L, respectively), the production of ARA was eventually reduced by approximately 49 % (Table 2).

**Table 1:** Fatty acid composition of different *M. alpina* strains grown in Kendrick medium for 168 h

Strain	Carbon source	Fatty acid content (%, w/w fatty acids)							
		C16:0	C18:0	C18:1	C18:2	C18:3	C20:3	C20:4	Total PUFAs
*M. alpina*	Glucose	13.9 ± 1.1^a^	11 ± 0.6^a^	10 ± 0.7^a^	17.1 ± 1.3^a^	2.7 ± 0.2^a^	1.9 ± 0.1^a,b^	33.4 ± 2.2^a^	55.2 ± 3.1^a^
*M. alpina*	Glycerol	15.1 ± 0.6^a^	14.4 ± 0.9^b,c^	12.1 ± 0.4^a,b^	11.4 ± 0.6^b^	1.3 ± 0.1^b,c^	1.2 ± 0.1^c,d^	34.5 ± 3.1^a^	48.5 ± 3.4^b^
MA-*gk*-1	Glycerol	15.3 ± 1.2^a^	14.8 ± 0.5^b,c^	11.4 ± 0.6^a,b^	13.5 ± 1.2^b,c,d^	2.1 ± 0.3^d^	1.5 ± 0.2^c,e^	32.4 ± 2.2^a^	49.6 ± 2.2^b^
MA-*gk*-2	Glycerol	14.6 ± 1.8^a^	12.9 ± 0.9^c^	13 ± 1.3^b^	14.3 ± 1.1^c,d^	2.1 ± 0.1^d^	1.5 ± 0.1^c,e^	31.4 ± 2.5^a^	49.3 ± 2.5^b^
MA-*gk*-3	Glycerol	15.3 ± 1.1^a^	14.3 ± 1.2^b,c^	11.9 ± 2.2^a,b^	15.8 ± 0.8^a,c^	1.9 ± 0.2^d,e^	1.1 ± 0.3^c,d^	29.7 ± 1.8^a^	48.4 ± 2.1^b^
MA-*g3pd1*-1	Glycerol	16.3 ± 0.7^a^	13.3 ± 1.2^b,c^	12.9 ± 0.8^b^	13.7 ± 0.4^b,c,d^	1.4 ± 0.2^b,c^	2.2 ± 0.2^a^	29.3 ± 1.3^a^	46.4 ± 1.7^b^
MA-*g3pd1*-2	Glycerol	15.7 ± 1.1^a^	15.3 ± 1.4^b^	13.7 ± 1.1^b^	11.7 ± 1.1^b^	1.9 ± 0.1^d,e^	1.6 ± 0.1^b,e^	30.3 ± 2.2^a^	45.6 ± 3.2^b^
MA-*g3pd1*-3	Glycerol	16.3 ± 1.2^a^	14.9 ± 1.1^b,c^	14.3 ± 1.5^b^	12.2 ± 0.6^b,d^	1.7 ± 0.4^b,d,e^	1.3 ± 0.1^c,d,e^	31.3 ± 1.2^a^	46.4 ± 2.6^b^
MA-*g3pd2*-1	Glycerol	16.1 ± 1.5^a^	14.8 ± 1.3^b,c^	13.5 ± 0.5^b^	14.5 ± 0.9 ^c,d^	1.1 ± 0.1^c^	1 ± 0.2^d^	30.1 ± 2.6^a^	46.8 ± 3.1^b^
MA-*g3pd2*-2	Glycerol	15.6 ± 2^a^	13.9 ± 0.9^b,c^	13.2 ± 0.8^b^	13.3 ± 1.3^b,c,d^	1.4 ± 0.1^b,c^	1.1 ± 0.2 ^c,d^	30.6 ± 0.7^a^	46.4 ± 1.6^b^
MA-*g3pd2*-3	Glycerol	15.5 ± 1.3^a^	14 ± 1.5^b,c^	13.1 ± 1.5^b^	12.7 ± 1.3^b,c,d^	1.6 ± 0.3^b,e^	1.4 ± 0.1^c,e^	31.2 ± 1.1^a^	46.9 ± 2.8^b^
MA-*g3pd3*-1	Glycerol	15.8 ± 1.6^a^	13.5 ± 1.2^b,c^	13.1 ± 1.1^b^	13.4 ± 0.7^b,c,d^	1.3 ± 0.1^b,c^	1.2 ± 0.1 ^c,d^	31.1 ± 3.1^a^	47 ± 2.8^b^
MA-*g3pd3*-2	Glycerol	15.6 ± 1.1^a^	13.6 ± 1.3^b,c^	13.3 ± 0.9^b^	13.7 ± 1.2^b,c,d^	1.6 ± 0.1^b,e^	1.2 ± 0.1^c,d^	30.9 ± 2.1^a^	47.4 ± 2.1^b^
MA-*g3pd3*-3	Glycerol	15.4 ± 1.4^a^	13.5 ± 1.1^b,c^	13.5 ± 1.1^b^	13.1 ± 0.8^b,d^	1.5 ± 0.1^b,c,e^	1.4 ± 0.1^c,e^	31.2 ± 2.2^a^	47.2 ± 2.5^b^

*M. alpina*: wild type *M. alpina*; MA-*gk*-1, MA-*gk*-2 and MA-*gk*-3: GK overexpressing strains; MA-*g3pd1*-1, MA- *g3pd1*-2 and MA-*g3pd1*-3: G3PD1 overexpressing strains; MA-*g3pd2*-1, MA-*g3pd2*-2 and MA-*g3pd2*-3: G3PD2 overexpressing strains; MA-*g3pd3*-1, MA-*g3pd3*-2 and MA-*g3pd3*-3: G3PD3 overexpressing strains. ^a,b,c,d,e^ mean the values within a row with different superscript letters were significantly different (*p* < 0.05) as analyzed by ANOVA

**Table 2:** Fatty acid production of different *M. alpina* strains grown in Kendrick medium for 168 h with glycerol as sole carbon source

Strain	Biomass (g/L)	Fatty acids (g/L)	ARA (mg/L)	Fatty acid yield (%)	ARA yield (%)
*M. alpina* (Glu*)	10.3 ± 0.6^a^	2.6 ± 0.2^a^	87.0 ± 6.1^a^	7.1 ± 0.5^a^	2.4 ± 0.2^a^
*M. alpina*	8.1 ± 0.3^b^	1.3 ± 0.1^b^	44.6 ± 2.9^b^	4.1 ± 0.2^b^	1.4 ± 0.1^b^
MA-*gk*-1	9.5 ± 1.1^a,c^	1.9 ± 0.2^c^	62.0 ± 4.5^c^	5.2 ± 0.4^c,d^	1.7 ± 0.1^c^
MA-*gk*-2	10.1 ± 0.5^a^	2.1 ± 0.2^d^	67.2 ± 4.7^c^	5.7 ± 0.4^c^	1.8 ± 0.1^c^
MA-*gk*-3	10 ± 0.6^a^	2.1 ± 0.1^c,d^	62.8 ± 3.2^c^	5.9 ± 0.5^c^	1.7 ± 0.1^c^
MA-*g3pd1*-1	8.5 ± 0.4^b,c^	1.5 ± 0.1^b^	43 ± 3.1^b^	4.3 ± 0.4^b^	1.3 ± 0.1^b^
MA-*g3pd1*-2	8.1 ± 0.5^b^	1.3 ± 0.1^b^	39.4 ± 2.9^b^	3.7 ± 0.4^b^	1.1 ± 0.1^d^
MA-*g3pd1*-3	8.4 ± 0.2^b^	1.3 ± 0.1^b^	40 ± 3.8^b^	3.8 ± 0.3^b^	1.2 ± 0.1^b,d^
MA-*g3pd2*-1	8.9 ± 0.7^b,c^	1.5 ± 0.1^b^	45 ± 2.4^b^	4.5 ± 0.3^b,d^	1.4 ± 0.1^b^
MA-*g3pd2*-2	8.7 ± 0.3^b,c^	1.5 ± 0.1^b^	45.3 ± 2.1^b^	4.6 ± 0.2^b,d^	1.4 ± 0.1^b^
MA-*g3pd2*-3	8.4 ± 0.5^b,c^	1.3 ± 0.1^b^	41.8 ± 2.6^b^	4.2 ± 0.3^b^	1.3 ± 0.1^b^
MA-*g3pd3*-1	8.6 ± 0.7^b,c^	1.3 ± 0.1^b^	41.7 ± 3.4^b^	4 ± 0.3^b^	1.2 ± 0.1^b,d^
MA-*g3pd3*-2	8.8 ± 0.4^b,c^	1.4 ± 0.1^b^	43.9 ± 4.1^b^	4.3 ± 0.2^b^	1.3 ± 0.1^b^
MA-*g3pd3*-3	8.8 ± 0.7^b,c^	1.4 ± 0.1^b^	44.5 ± 3.1^b^	4.3 ± 0.3^b^	1.3 ± 0.1^b^

*M. alpina*: wild type *M. alpina*; MA-*gk*-1, MA-*gk*-2 and MA-*gk*-3: GK overexpressing strains; MA-*g3pd1*-1, MA-*g3pd1*-2 and MA-*g3pd1*-3: G3PD1 overexpressing strains; MA-*g3pd2*-1, MA-*g3pd2*-2 and MA-*g3pd2*-3: G3PD2 overexpressing strains; MA-*g3pd3*-1, MA-*g3pd3*-2 and MA-*g3pd3*-3: G3PD3 overexpressing strains. ^a,b,c,d^ Mean the values within a row with different superscript letters were significantly different (*p* < 0.05) as analyzed by ANOVA. * Cultured with glucose as carbon source

**Fig. 3:**
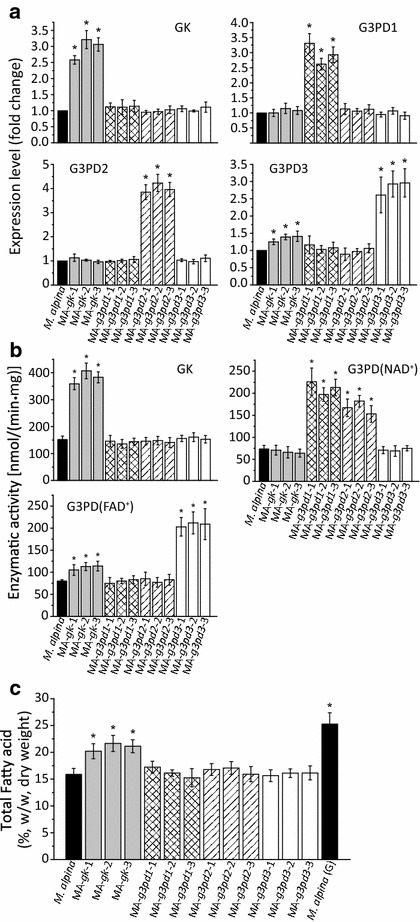
Overexpression of GK and G3PDs in *M. alpina.* The expression level (**a**), enzymatic activity (**b**) and total fatty acid level (**c**) in *M. alpina* strains were analyzed after overexpressing GK and G3PDs. *M. alpina* (*black* *bars*): wild type *M. alpina*; MA-*gk*-1, MA-*gk*-2, MA-*gk*-3 (*gray bars*): GK-overexpressing *M. alpina* strain; MA-*g3pd1*-1, MA- *g3pd1*-2, MA-*g3pd1*-3 (*cross-hatched bars*): G3PD1-overexpressing *M. alpina* strain; MA-*g3pd2*-1, MA-*g3pd2*-2, MA-*g3pd2*-3 (*striped bars*): G3PD2-overexpressing *M. alpina* strain; MA-*g3pd3*-1, MA-*g3pd3*-2, MA-*g3pd3*-3 (*white bars*): G3PD3-overexpressing *M. alpina* strain; *M. alpina* (G): *M. alpina* growing with glucose as carbon source. Strains were cultured in 500 mL shaking flasks containing 200 mL Kendrick medium supplemented with 50 g/L glycerol for 168 h at 200 rpm. Three independent experiments were performed, and the* error bars* represent standard deviations. *p < 0.05 compared to wild type

To increase the glycerol assimilation efficiency in *M. alpina*, the genes encoding GK and three isoforms of G3PD were independently overexpressed by introducing an additional copy of each gene under a homologous His 550 promoter. The inserted expression cassettes in the genome were identified by PCR with two pairs of promoter and terminator specific primers [20, 28]. *M. alpina* strains were analyzed after being cultured for 168 h in sterilized flasks containing 200 mL Kendrick medium, a nitrogen-limited medium commonly used for studying fatty acid accumulation in oleaginous microorganisms [29]. The transcript levels of all overexpressed genes in each overexpressing strain significantly increased by approximately twofold to fourfold compared to wild-type strain (Fig. 3a). The expression of G3PD3 gene (*g3pd3*) was also found to be up-regulated in GK gene (*gk*) overexpressing strains; this may be due to an increase in the substrate (glycerol-3-phosphate) generated by GK. The same trend was also detected in terms of the enzymatic activity of GK and G3PDs (Fig. 3b). When *gk* was overexpressed, the fatty acid content significantly increased by over 35 % compared to control. By contrast, none of the G3PD overexpressing strains exhibited any improvement in fatty acid content (Fig. 3c).

**Fig. 4:**
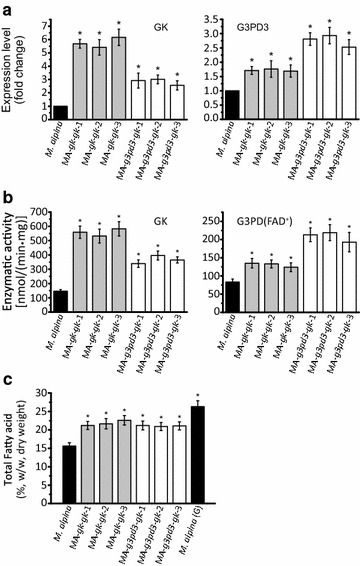
Double-overexpression of GK and co-overexpression of GK and G3PD3 in *M. alpina.* The expression level (**a**), enzymatic activity (**b**) and total fatty acid level (**c**) in *M. alpina* strains were analyzed after double-overexpression of GK and co-overexpression of GK and G3PD3. *M. alpina* (*black bars*): wild type *M. alpina*; MA-*gk*-*gk*-1, MA-*gk*-*gk*-2, MA-*gk*-*gk*-3 (*gray bars*): GK double-overexpressing *M. alpina* strain; MA-*g3pd3*-*Gk*-1, MA-*g3pd3*-*Gk*-2, MA-*g3pd3*-*Gk*-3 (*white bars*): GK and G3PD3 co-overexpressing *M. alpina* strain; *M. alpina* (G): *M. alpina* growing with glucose as carbon source. Strains were cultured in 500 mL shaking flasks containing 200 mL Kendrick medium supplemented with 50 g/L glycerol for 168 h at 200 rpm. Three independent experiments were performed, and the *error bars* represent standard deviations. *p < 0.05 compared to wild type

Next, we wondered if increasing further GK expression level by augmenting its copy number could improve the assimilating efficiency of glycerol in *M.alpina*. However, further increased expression of GK may now cause G3PD to be a limiting step. Therefore, *gk* was double-introduced into *M. alpina* or co-introduced with *g3pd3*. The transcript levels of *gk* and *g3pd3* in each double- or co- overexpressing strain were significantly higher than before (Fig. 4a), as well as their enzymatic activities (Fig. 4b). However, the fatty acids were not further accumulated compared to strains with single gene overexpression (Fig. 4c). This suggests that there are other limiting factors that affect fatty acid synthesis when *M. alpina* is grown with glycerol as carbon source.

### NADPH levels in *M. alpina* with different carbon sources

**Fig. 5:**
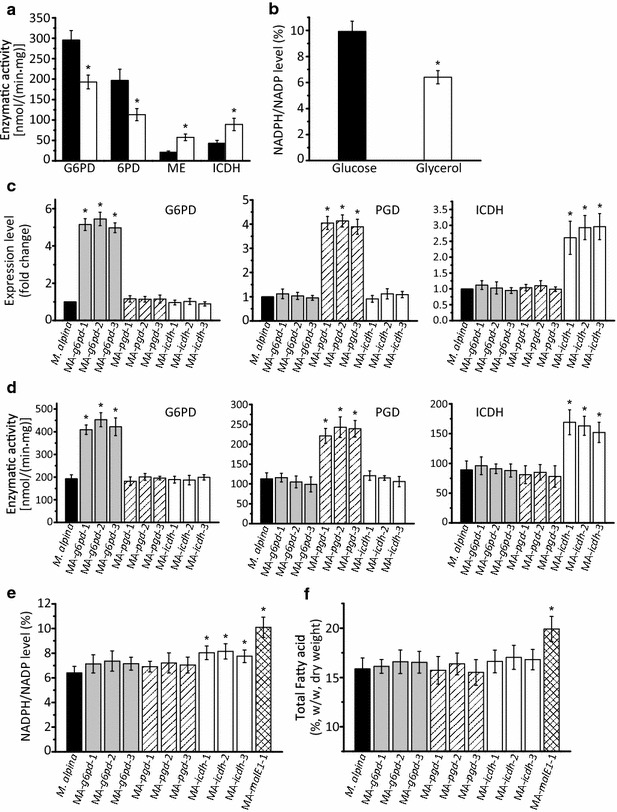
Comparison of the enzymatic activity (**a**) and NADPH level (**b**) between *M. alpina* cultures growing in the presence of glucose (*black bars*) and glycerol (*white bars*). The expression level (**c**), enzymatic activity (**d**), NADPH level (**e**) and total fatty acid level (**f**) in *M. alpina* strains were analyzed after overexpressing G6PD, PGD and ICDH. *M. alpina* (*black bars*): wild type *M. alpina*; MA-*g6pd*-1, MA-*g6pd* -2, MA-*g6pd* -3 (*gray bars*): G6PD-overexpressing *M. alpina* strains; MA-*Pgd*-1, MA-*Pgd*-2, MA-*Pgd*-3 (*slash bars*): PGD-overexpressing *M. alpina* strains; MA-*icdh*-1, MA-*icdh*-2, MA-*icdh*-3 (*white bars*): ICDH-overexpressing *M. alpina* strain; MA-*malE1*-1 (*cross-hatched bars*): ME1-overexpressing *M. alpina* strain (previously constructed). Strains were cultured in 500 mL shaking flasks containing 200 mL Kendrick medium supplemented with 50 g/L glycerol for 168 h at 200 rpm. Three independent experiments were performed, and the* error bars* represent standard deviations. *p < 0.05 compared to wild type

NADPH is the reducing power for fatty acid synthesis and has been proved as the decisive factor that determines fatty accumulation in oleaginous fungi [30, 31]. Recently, the PPP was identified to be a key step during fatty acid synthesis, mainly by providing NADPH [18, 19]. We noted that, in the absence of glucose, the activities of glucose-6-phosphate dehydrogenase (G6PD, EC 1.1.1.49) and 6-phosphogluconate dehydrogenase (PGD, EC 1.1.1.44) were significantly decreased and remained at moderate levels (Fig. 5a). Accordingly, cell NADPH level decreased to a relatively low level (Fig. 5b). This may lead to another bottleneck when the assimilation efficiency of glycerol is adequately improved. ME has already been demonstrated to be an important NADPH source for fatty acid synthesis [20]. Alternatively, ICDH is another potential NADPH supplier that needs to be further analyzed in order to determine its role in fatty acid synthesis compared to ME [21].

### Overexpression of G6PD, PGD and ICDH in *M. alpina*

To provide a better NADPH source in *M. alpina*, the genes encoding G6PD, PGD and ICDH (identified or speculated as critical steps for fatty acid synthesis) were overexpressed independently [18, 21]. The transcript level and enzymatic activity of each of three strains that overexpressed a different single genes were analyzed, with wild-type *M. alpina* as control. All the overexpressing strains exhibited improved transcript level and enzymatic activity, respectively (Fig. 5c and d). The cytosolic NADPH level was further analyzed, along with the ME1 gene (*malE1*) overexpressing strain MA-*malE1*-1 (Fig. 5e; Additional file 1: Fig S1A). In the *icdh* overexpressing strains, NADPH ratios were significantly improved compared with control, but not as high as in MA-*malE1*-1 (Fig. 5e). Whereas, the NADPH contents of wet weight (WW) were not significantly improved in *icdh* overexpressing strains (Additional file 1: Fig S1A). By contrast, the NADPH levels of the G6PD and PGD overexpressing strains were apparently not affected, which may be due to substrate insufficiency caused by the absence of glucose in the medium (Fig. 5e; Additional file 1: Fig S1A). Accordingly, fatty acid accumulation was also differentially affected by the increased supply of NADPH. As shown in Fig. 5f, the improvement of fatty acid content in ICDH gene (*icdh*) overexpressing strains reached approximately 17 % of dry cell weight (DCW), compared with 20 % of the MA-*malE1*-1 strain. The results indicate that ME1 is a better NADPH supplier than G6PD, PGD and ICDH during fatty acid synthesis in *M. alpina* cultured with glycerol as carbon source.

### Co-overexpression of GK and ME1 in *M. alpina*

**Fig. 6:**
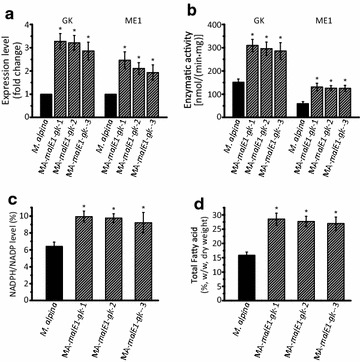
Co-overexpression of ME1 and GK in *M. alpina.* The expression level (**a**), enzymatic activity (**b**), NADPH level (**c**) and total fatty acid level (**d**) in *M. alpina* strains were analyzed after co-overexpressing ME1 and GK. *M. alpina* (*black bars*): wild type *M. alpina*; MA-*malE1*-*gk*-1 (*striped bars*): ME1 and GK co-overexpressing *M. alpina* strain. Strains were cultured in 500 mL shaking flask containing 200 mL Kendrick medium supplemented with 50 g/L glycerol for 168 h at 200 rpm. Three independent experiments were performed, and the *error bars* represent standard deviations. *p < 0.05 compared to wild type

Based on the results above, we deduced that fatty acid accumulation in *M. alpina* cultured with glycerol was affected by both the assimilation of glycerol and the supply of NADPH, which could be substantially improved by GK and ME1. Therefore, the genes encoding GK and ME1 were co-expressed to further increase PUFA production compared to single gene overexpression. The transcript level and enzymatic activity of GK and ME1 in three co-overexpressing strains were increased twofold to threefold (Fig. 6a and b), and the NADPH level was also improved owing to the overexpression of ME1 (Fig. 6c; Additional file 1: Fig S1B). The fatty acid content increased by approximately 80 % compared to wild type control after culture in Kendrick medium for 168 h in shaking flasks (Fig. 6d).

### Batch fermentation of MA-*malE1*-*gk*-1 on glycerol

**Fig. 7:**
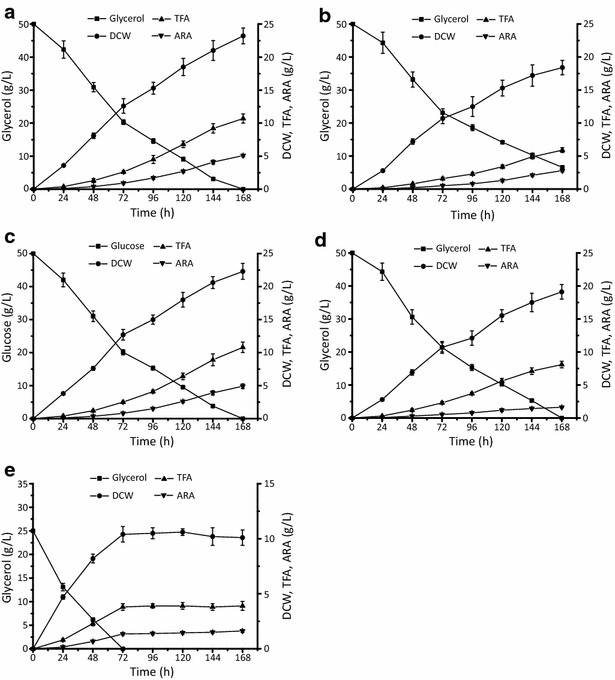
The time course of residual carbon source concentrations, total fatty acid (TFA), dry cell weight (DCW) and arachidonic acid (ARA) in batch fermentation of *M. alpina* strains. **a** The co-overexpressing strain MA-*malE1*-*gk*-1 cultured with 50 g/L pure glycerol. **b** Wild type *M. alpina* cultured with 50 g/L pure glycerol. **c** Wild type *M. alpina* cultured with 50 g/L glucose. **d** The co-overexpressing strain MA-*malE1*-*gk*-1 cultured with 50 g/L raw glycerol. **e** The co-overexpressing strain MA-*malE1*-*gk*-1 cultured with 25 g/L raw glycerol. Three independent experiments were performed, and the *error bars* represent standard deviations

Batch fermentations were carried out in order to systematically analyze fatty acid production of the engineered *M. alpina* strain MA-*malE1*-*gk*-1. The total fatty acid (TFA) and ARA production reached 10.7 ± 0.6 and 4.9 ± 0.3 g/L, respectively (Fig. 7a, Table 3), after being fermented with 50 g/L glycerol for 168 h. Compared with wild-type *M.* *alpina*, the TFA content (% of DCW) and production (g/L culture) of MA-*malE1*-*gk*-1 increased by approximately 44 % and 81 %, respectively (Fig. 7b) and reached levels comparable to those observed when cultured with glucose (Fig. 7c).

**Table 3:** Fatty acid production of different *M. alpina* strains in batch fermentation

Strain	Carbon source (g/L)	Time (h)	Biomass (g/L)	Fatty acids (g/L)	ARA (g/L)	Fatty acid yield (%)	ARA yield (%)
Batch							
*M. alpina*	Glu (50)	168	22.3 ± 1.8^a^	10.2 ± 0.7^a^	4.6 ± 0.2^a^	20 ± 1.7^a^	9.2 ± 0.4^a^
*M. alpina*	Gly (50)	168	18.4 ± 1.1^b^	5.9 ± 0.2^b^	2.6 ± 0.2^b^	13.6 ± 0.7^b,c^	6 ± 0.3^b^
MA-*malE1*-*gk*-1	Gly (50)	168	23.2 ± 2.1^a^	10.7 ± 0.6^a^	4.9 ± 0.3^a^	21.4 ± 1.5^a^	9.8 ± 0.5^a^
MA-*malE1*-*gk*-1	Raw Gly (50)	168	19.1 ± 1.3^b^	8.1 ± 0.5^c^	1.6 ± 0.1^c^	16.2 ± 0.8^d^	3.3 ± 0.1^c^
MA-*malE1*-*gk*-1	Raw Gly (25)	168	10.1 ± 0.9 ^c,d^	3.9 ± 0.6^d^	1.6 ± 0.2^c^	15.6 ± 1.2^b,d^	6.5 ± 0.4^b^
Repeated Batch							
MA-*malE1*-*gk*-1							
Round I	Raw Gly (25)	72	10.6 ± 0.6^c^	3.9 ± 0.1^d^	1.4 ± 0.1^c,d^	15.6 ± 1.1^b,d^	5.6 ± 0.2^d^
Round II	Raw Gly (25)	72	10.8 ± 0.5^c^	3.6 ± 0.2^d,e^	1.4 ± 0.1^c,d^	14.4 ± 1.2^b,c,d^	5.4 ± 0.3^d,e^
Round III	Raw Gly (25)	72	10.1 ± 0.9^c,d^	3.3 ± 0.2^d,e^	1.2 ± 0.1^d^	13.2 ± 0.6^c^	4.8 ± 0.3^e^
Round IV	Raw Gly (25)	72	8.2 ± 0.8^d^	2.9 ± 0.2^e^	0.9 ± 0.1^e^	12.4 ± 0.9^c^	4 ± 0.2^f^

*M. alpina*: wild type *M. alpina*; MA-*malE1*-*gk*-1: ME1 and GK co-overexpressing strain; *Glu* glucose; *Gly* Glycerol; *Raw Gly* Raw Glycerol. ^a,b,c,d,e,f^ mean the values within a row with different superscript letters were significantly different (*p* < 0.05) as analyzed by ANOVA

As purification cost is relatively expensive, directly using raw glycerol as a substrate to produce high value added products has becoming the most reasonable way to make use of waste glycerol. The performance of the MA-*malE1*-*gk*-1 strain co-overexpressing *malE1* and *gk* on 50 g/L raw glycerol as substrate was evaluated (Fig. 7d). The production of TFA and ARA was significantly affected by the impurity of raw glycerol and decreased to 8.1 ± 0.5 and 1.6 ± 0.1 g/L, respectively (Fig. 7d, Table 3). Notably, the mycelium morphology was found to be basically filamentous, which may increase the medium viscosity and affect the absorption of the substrate as well as the accumulation of ARA. As a result, the ARA content only reached 20 % of TFA after being fermented for 168 h (Table 4). Thus, the raw glycerol in the fermentation medium was reduced to 25 g/L and was assimilated and exhausted within 72 h (Fig. 7e). It is noteworthy that, during the first 24 h, the lag phase was shortened and the cells grew faster than when fermented with 50 g/L carbon source (Fig. 7e).

**Table 4:** Fatty acid composition of different *M. alpina* strains in batch fermentation

Strain	Carbon source (g/L)	Time (h)	Fatty acid content (%, w/w fatty acids)						
			C16:0	C18:0	C18:1	C18:2	C18:3	C20:3	C20:4
Batch									
*M. alpina*	Glu (50)	168	9.4 ± 0.6^a^	6.2 ± 0.4^a^	15.1 ± 1.1^a^	6.5 ± 0.3^a^	4.1 ± 0.2^a^	5.1 ± 0.3^a^	45.1 ± 1.2^a^
*M. alpina*	Gly (50)	168	11.2 ± 0.5^a,b^	12.6 ± 0.8^b^	13.6 ± 0.9 ^a,b^	3.8 ± 0.3^b^	3.1 ± 0.1^b^	1.5 ± 0.1^b^	44.1 ± 2.5^a^
MA-*malE1*-*gk*-1	Gly (50)	168	10.2 ± 0.4^a^	9.6 ± 0.6^b^	14.1 ± 1.1^a,b^	4.1 ± 0.2^b^	4.2 ± 0.1^b^	1.5 ± 0.1^b,c^	45.8 ± 1.3^a^
MA-*malE1*-*gk*-1	Raw Gly (50)	168	17.9 ± 1.2^c^	11.7 ± 0.6^c,d^	25.9 ± 2.3^c^	9.5 ± 0.6^c^	5.1 ± 0.2^c^	2.1 ± 0.1^d^	20.1 ± 1.9^b^
MA-malE1-*gk*-1	Raw Gly (25)	168	12.9 ± 0.5^b^	8.6 ± 0.5^d^	11 ± 0.8^d^	9.7 ± 1.1^c^	5.5 ± 0.4^c^	2.3 ± 0.2^d^	41.5 ± 1.7^a^
Repeated Batch									
MA-*malE1*-*gk*-1									
Round I	Raw Gly (25)	72	17.6 ± 1.3^c^	7.6 ± 0.6^e^	14.3 ± 1.2^a,b^	9.6 ± 0.5^c^	5.3 ± 0.3^c^	1.3 ± 0.1^b,c^	36.2 ± 2.6^d^
Round II	Raw Gly (25)	72	17.1 ± 1.6^c^	9.5 ± 0.3^c,d^	12.4 ± 1.5^b,d^	8.2 ± 0.4^d^	6.7 ± 0.2^d^	1.4 ± 0.1^b^	37.8 ± 3.5^c,d^
Round III	Raw Gly (25)	72	15.1 ± 1^d^	8.9 ± 0.2^c,d^	18.9 ± 1.1^e^	7.6 ± 0.4^d^	5.6 ± 0.2^c^	1.3 ± 0.1^b,c^	36.7 ± 1.9^c,d^
Round IV	Raw Gly (25)	72	18.7 ± 1.3^c^	9.8 ± 0.8^c^	18.4 ± 0.8^e^	7.3 ± 0.6^a,d^	5.1 ± 0.2^c^	1.1 ± 0.1^c^	29.7 ± 1.7^e^

*M. alpina*: wild type *M. alpina*; MA-*malE1*-*gk*-1: ME1 and GK co-overexpressing strain; *Glu* glucose; *Gly* Glycerol; *Raw Gly* Raw Glycerol. ^a,b,c,d,e^ mean the values within a row with different superscript letters were significantly different (*p* < 0.05) as analyzed by ANOVA

**Fig. 8:**
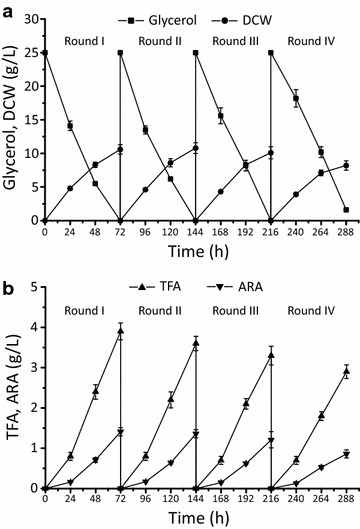
Time course of **a** residual carbon source concentrations, dry cell weight (DCW); **b** total fatty acid (TFA) and arachidonic acid (ARA) in repeat batch fermentation of MA-*malE1*-*gk*-1. 10 % of the culture was used as the inoculum for the next round fermentation by supplementing 3.6 L of fresh medium at the end of each round for the first three fermentations

In view of the results, the repeat batch fermentation process that keeps the glycerol and exogenous fatty acids in the medium at a relative low level was applied to improve the production efficiency of fatty acids including ARA (Fig. 8). After a round of fermentation, 10 % of fermented broth was retained as the seed for the next round fermentation, supplemented with 3.6 L fresh medium. The four-round repeat batch fermentation lasted 288 h and consumed 100 g raw glycerol in total. The repeat batch was ended at round IV, owing to the decreased DCW and ARA production. From rounds I to IV, TFA reached over 30 % of the DCW, which was 10.6 ± 0.6, 10.8 ± 0.5, 10.1 ± 0.9 and 8.2 ± 0.8 g/L (Fig. 8a and b, Table 3). The ARA content ranged between 35 and 40 % of TFA with an average yield of 52.2 ± 1.9 mg/g glycerol. Compared with 32.6 ± 1.4 mg/g when fermented with 50 g/L raw glycerol, the repeat batch method improved the yield of ARA by over 60 % (Figs. 7d and 8b, Table 3). We also noted the DCW and ARA production was increasingly affected from rounds I to IV (Tables 3 and 4). This may be due to mycelium morphology changes from feather-like to a tight pellet, affecting the transfer of nutrients and oxygen [32, 33].

## Discussion

In this study, the genes encoding for GK and G3PD in *M. alpina* were overexpressed in order to determine their effect on fatty acid production with glycerol as carbon source (Fig. 3). However, only GK overexpression significantly improved TFA content, by 35 % compared to the wild type control (Fig. 3c). By contrast, independent overexpression of three G3PDs had no effect on fatty acid accumulation. In human adipose tissue, the NAD^+^ dependent G3PD was reported to catalyze reaction in both directions with a similar efficiency [34]. It is possible that G3PD1 and G3PD2 in *M. alpina* have similar characteristics, and thus overexpression of these genes would not increase glycerol assimilation (Fig. 3b). In case of the FAD^+^ dependent G3PD3, it favors the formation of DHAP [35]. Interestingly, endogenous G3PD3 expression seems to be regulated by its substrate level. When GK was overexpressed, the transcription of G3PD3 was also increased, as well as its enzymatic activity (Fig. 3a and b). In addition, overexpression alone or in combination with GK did not significantly affect TFA accumulation. These results reinforce the idea that GK but not G3PD is the limiting step during glycerol assimilation in *M. alpina*.

The supply of cytosolic NADPH, which has been demonstrated to be critical for fatty acid accumulation in oleaginous fungi, may constitute another limited factor [20]. There are four main cytosolic NADPH sources, i.e. G6PD, PDG, ME and ICDH, their roles during fatty acid synthesis need to be further evaluated. When *M. alpina* cultured in glycerol, the PPP was significantly suppressed (Fig. 5a). As a result, the NADPH level was insufficient to sustain a high rate of fatty acid syntheses (Fig. 5b). Overexpression of G6PD and PGD neither significantly improved the NADPH level nor the fatty acid accumulation. This may be because, in the absence of glucose, there is a low level of substrate for the PPP. Overexpression of ICDH increased NADPH level, but the extent was too slight to have an impaction on fatty acid accumulation (Fig. 5e). This may due to the insufficient cytosolic isocitrate generation that relies on a partial reverse of TCA for lipogenesis [36]. But this catalytic flux was indicated not as persisted as its forward direction [37]. Overexpression of ME1 had the most significant effect on NADPH level and fatty acid synthesis. Subsequently, ME1 was co-overexpressed with GK in the MA-*malE1*-*Gk*-1 strain of *M. alpina*. In the presence of pure glycerol the TFA levels increased by 81 % compared to the wild-type control. When *M.alpina* grow with glycerol as sole carbon source, the role of ME became more prominent due to the inability of PPP to provide NADPH needed for fatty acid synthesis.

When raw glycerol is directly used as carbon source, its impurity will affect *M. alpina* proliferation and in growing and fatty acid synthesis, especially the PUFAs [25, 38]. This suppression may probably be due to the exogenous fatty acids that affects the activities of desaturases and elongases [25]. In *M. alpina*, ARA is the major product and its synthesis is suppressed by these impurities. Therefore, repeated batch method was applied to alleviate the inhibitory effect of raw glycerol, and to shorten the seed cultivation process [39]. The ARA yield improved by over 60 % and the batch was ended after round IV.

## Conclusions

In conclusion, we reported a bioengineering strategy, by improving the upstream assimilation and NADPH supply, for oleaginous fungi to efficiently accumulate fatty acid with glycerol as carbon source. The co-overexpression of GK and ME1 improved fatty acid accumulation by 81 % in *M. alpina* when grown with pure glycerol. When the repeat batch method was applied to relieve the inhibitory effect of high raw glycerol concentration, ARA yield was improved by 60 %. Therefore, our work represents a significant step toward a high value added strategy of utilizing biodiesel-derived waste and presents new engineering insight for the production of other compounds with raw glycerol as carbon source.

## Methods

### Strains and culture media

*Mortierella alpina* ATCC 32,222 was cultured on potato dextrose agar (PDA) medium and its uracil auxotrophic strain CCFM 501 [20] was maintained on GY medium, consisting of 30 g/L glucose, 5 g/L yeast extract, 2 g/L KNO_3_, 1 g/L NaH_2_PO_4_ and 0.3 g/L MgSO_4_·7H_2_O, containing 5-fluoroorotic acid (5-FOA, 0.5 mg/mL) and uracil (0.05 mg/mL). *Escherichia coli* top 10 was cultivated at 37 °C on LB agar plates and used for plasmid construction. *Agrobacterium tumefaciens* C58C1 was cultivated at 28 °C on YEP medium consisting of 10 g/L tryptone, 10 g/L yeast extract and 5 g/L NaCl and used as T-DNA donor for fungal transformation. The composition of the minimal medium (MM) and induction medium (IM) were previously described [40]. The composition of the synthetic complete (SC) medium, which was used for the positive selection of the transformants, was described before [28]. Kendrick medium [29] was used for the fatty acid analysis in flask culture of *M. alpina* strains and consisted of 50 g/L glucose (glycerol), 2.0 g/L diammonium tartrate, 7.0 g/L KH_2_PO_4_, 2.0 g/L Na_2_HPO_4_, 1.5 g/L MgSO_4_·7H_2_O, 1.5 g/L yeast extract, 0.1 g/L CaCl_2_·2H_2_O, 8 mg/L FeCl_3_·6H_2_O, 1 mg/L ZnSO_4_·7H_2_O, 0.1 mg/L CuSO_4_·5H_2_O, 0.1 mg/L Co(NO_3_)_2_·6H_2_O and 0.1 mg/L MnSO_4_·5H_2_O, pH 6.0. The medium used for the batch fermentation consisted of 50 g/L glucose (glycerol), 5 g/L yeast extract, 1.0 g/L KH_2_PO_4_, 0.25 g/L MgSO_4_·7H_2_O, 10 g/L KNO_3_. Raw glycerol with 80 % purity was added to reach 50 g/L glycerol concentration.

### Fermentation conditions

Batch fermentations of *M. alpina* strains were carried out in a 7.5-L fermenter (BioFlo/CelliGen 115, New Brunswick Scientific, Edison, NJ, USA). The incubation protocols were as previously described [41]. *M. alpina* was cultured on PDA or GY plates for 3 weeks at 28 °C. Five mL liquid Kendrick medium was added and the spores were scraped with a sterile loop. Three mL of the spore suspension was inoculated into 50 mL Kendrick medium in a 250 mL flask and cultured at 28 °C for 5 days with shaking at 200 rpm. Cultures were blended for eight pulses using a Braun hand blender with 5 s/pulse. Inoculate 0.3 g wet into 50 mL Kendrick medium in a 250 mL flask and cultured at 28 °C for 5 days with shaking at 200 rpm. The above step was repeated once to make the fungal culture in proliferative phase. The proliferative phase cultures were inoculated at 10 % (v/v) into Kendrick medium to form 4 L culture in a 7.5-L fermenter. The temperature was held at 28 °C and the agitation rate was 500 rpm. Air flow rate was maintained at 0.5 vvm, and the pH was maintained at 6.0 by the automatic addition of 2 M of KOH and HCl.

### Construction of T-DNA binary vector

The *gk*, G3PD1 gene (*g3pd1*), G3PD2 gene (*g3pd2*), *g3pd3*, G6PD gene (*g6pd*), PGD gene (*pgd*) and *icdh* were amplified from the *M. alpina* cDNA with the primer pairs listed in Additional file 1: Table S1. Genes were ligated into the pGEM-T easy vector (Promega, Madison, WI, USA) followed by a sequence analysis on ABI PRISM 3730. After being digested with appropriate restrict enzymes, genes were ligated into the binary vector pBIG2-ura5 s-ITs [28] to construct single-gene expression vector. Genes were driven by a homologous constitutive His 550 promoter that was commonly used for gene overexpression in *M. alpina*. The expression of His 550 promoter might keep decreasing after nitrogen exhaustion [20]. Expression cassette was amplified with primer pair InFusF/InFusR and ligated into XbaI digested single gene expression vector using In-Fusion HD Cloning Kit (Clontech Laboratories, Mountain View, CA, USA) to construct co-expression vector.

### *Agrobacterium tumefaciens*-mediated transformation (ATMT)

*Agrobacterium tumefaciens*-mediated transformation was performed following a previously described protocol [20]. *M. alpina* CCFM 501 spores were harvested from GY agar medium cultures (supplemented with 0.05 g/mL uracil). *A. tumefaciens* C58C1 was electro transformed with the corresponding binary vector as previously described [42] and the transformants were isolated on YEP agar plates supplemented with 100 μg/mL kanamycin and 100 μg/mL rifampicin, followed by PCR confirmation of positive transformants. After an induction culture at 28 °C in liquid IM to an OD_600nm_ of 0.8–1.2, 100 μL of the *A. tumefaciens* suspension was mixed with an equal volume of spore suspension (10^8^/mL) and then spread on cellophane membranes, which were placed on a solid cocultivation medium (IM containing 5 mM glucose). The plates were incubated at 23 °C for 24–72 h in a dark incubator and transferred to uracil-free SC plates (containing 50 μg/mL cefotaxime and 50 μg/mL spectinomycin to inhibit the growth of bacteria), then incubated at 25–30 °C until colonies appeared. The mycelium was transferred to fresh SC plates, and the procedure was repeated three times to obtain stable strains. These stable transformed strains were maintained for further analysis. All experiments were carried out in triplicate.

### Genomic DNA preparation

*M.alpina* strains were cultivated in GY liquid medium at 28 °C for 4 days at 200 rpm. Mycelia were harvested and washed twice with sterile water then frozen immediately in liquid nitrogen. Genomic DNA of *M.alpina* was extracted as described previously [41].

### RT-qPCR analysis

The primer pairs used for RT-qPCR are shown in Additional file 1: Table S1. Total RNA was isolated from *M. alpina* and reverse-transcribed with the PrimeScript RT reagent kit (Takara Bio, Japan) according to the manufacturer’s instructions. RT-qPCR was performed on the ABI-Prism 7900 sequence detection system (Applied Biosystems, CA) with the Power SYBR Green PCR Master Mix (Applied Biosystems, CA). Twenty-microliter reaction mixtures composed of 10 µL of SYBR Green PCR Master Mix, 0.5 µL of each primer, 8 µL of distilled water, and 1 µL of DNA template or distilled water as negative control were prepared. The PCR cycling conditions were 50 °C for 2 min, 95 °C for 10 min, followed by 40 cycles of amplification at 95 °C for 15 s and 60 °C for 30 s. The expression of the internal control gene (18S rRNA) was used as the normalization standard for gene expression. All of the samples were measured in triplicate.

### Determination of enzymatic activities

Mycelium was harvested by filtration, then frozen and ground in liquid nitrogen and suspended in the previously described extraction buffer [28]. The activity of ME, ICDH, G6PD and PGD was determined as described before [20, 43, 44]. GK and FAD^+^ dependent G3PD activity was determined as described previously [45, 46]. NAD^+^ dependent G3PD activity was determined as described previously [47].

### NADP and NADPH quantification

Essentially as described previously [20], samples were rapidly collected and frozen with liquid nitrogen, lyophilized and ground in liquid nitrogen. The NADP and NADPH levels were analyzed using the NADP/NADPH Quantification Colorimetric Kit (BioVision, California, USA) according to the manufacturer’s instructions.

### Fatty acid methyl ester (FAME) analysis

For fatty acid analysis, the *M.alpina* mycelia were collected and lyophilized. Approximately 20 mg of mycelia were used for each lipid extraction. Fatty acid analysis was performed essentially as described previously [41]. FAMEs were subsequently analyzed by GC–MS (GC-2010 Plus; MS-QP2010 Ultra, Shimadzu Co., Kyoto, Japan) with a 30 m × 0.25 mm Rtx-Wax column (film thickness 0.25 µm) with the temperature program: 40 °C for 5 min, ramp to 120 °C at 20 °C per min, then ramp to 190 °C at 5 °C per min, and hold for 5 min, then ramp to 220 °C at 5 °C per min, and finally hold for 17 min. Helium was used as the carrier gas. Fatty acid quantification was carried out using peak-height area integrals. A 37-component FAME mix was used for qualitative analysis. Pentadecanoic acid and heneicosanoic acid were used as internal standards to quantify the fatty acid methyl esters with aliphatic chain ≤18 and >18, respectively. All experiments were carried out in triplicate.

### Statistical analysis

All experiments were carried out in triplicate, and the means and standard errors were calculated. SPSS 20 was used for one-way analysis and canonical correlation analysis, and the significant differences (*P* < 0.05) were determined by the least significant difference test.

## Additional file

10.1186/s12934-015-0392-4 Primers used in this study.

## Abbreviations

GK: glycerol kinase
G3PD: glycerol-3-phosphate dehydrogenase
ME: malic enzyme
G6PD: glucose-6-phosphate dehydrogenase
PGD: 6-phosphogluconate dehydrogenase
ICDH: isocitrate dehydrogenase
ARA: arachidonic acid
PUFAs: polyunsaturated fatty acids
DHAP: dihydroxyacetone phosphate
ATMT: *Agrobacterium tumefaciens*-mediated transformation
GAP: glyceraldehyde 3-phosphate
PEP: phosphoenolpyruvate
2-KG: 2-ketoglutarate
TPI: triose phosphate isomerase
GAPDH: glyceraldehyde-3-phosphate dehydrogenase
PK: pyruvate kinase
RPI: ribose-5-phosphate isomerase
TK: transketolase
ACLATP: citrate synthase
MDH: malate dehydrogenase
ACC: acetyl-CoA carboxylase
FASn: fatty acid synthase
TFA: total fatty acids
DCW: dry cell weight
