# Supplementary material for: Metabolic engineering of Mortierella alpina for arachidonic acid production with glycerol as carbon source
Source: Microb Cell Fact. 2015 Dec 23;14:205. doi: 10.1186/s12934-015-0392-4 (PMC4690419; doi:10.1186/s12934-015-0392-4)
Supplement: Supplementary file 1 — 10.1186/s12934-015-0392-4 Primers used in this study. [file 12934_2015_392_MOESM1_ESM.docx]

**Table S1** Primers used in this study.

| Primer | Sequence (5' -3')^a^ | Description |
| --- | --- | --- |
| **Primers used for PCR amplification** | |  |
| GKF | ATACCCAAGCTTATATGCCCTCCTTCATTGGCG | GK amplification |
| GKR | TAATTCCCCCGGGCTACTCTGCCAAAGTCTTTGCGAT |  |
| G3PD1F | GCACGGGGTACCATGTGGCGCCGAATCTCAAC | G3PD1 amplification |
| G3PD1R | GCTCCCCCCGGGCTATGCTCCACCACCGCTGC |  |
| G3PD2F | GCACGGGGTACCATGTCGGAAAAAGTAGCAATCATC | G3PD2 amplification |
| G3PD2R | GCTCCCCCCGGGTCAAATATCCTCCACAATTCTAATG |  |
| G3PD3F | GCACGGGGTACCATGTGGCGCCGAATCTCAAC | G3PD3 amplification |
| G3PD3R | GCTCCCCCCGGGCTATGCTCCACCACCGCTGC |  |
| G6PDF | GCACGGGGTACCATGTCTGAGAAGAAGAAGCATCTTT | G6PD amplification |
| G6PDR | GCTCCCCCCGGGTTAATGGTCAGTCCTTGTGTCCT |  |
| PGDF | GCACGGGGTACCATGAACGACAATGGCTACACC | PGD amplification |
| PGDR | GCTCCCCCCGGGTTAAGCAAGGTAGGTGGTCGAG |  |
| ICDHF | ATACCCAAGCTTGAATGCTTGCCAACAAAATCAACG | ICDH amplification |
| ICDHR | ATACCCGAGCTCTTAAACGGTGCGCTTCTTCTGC |  |
| InFusF | CTCTCCTATGAGTCGTTTACCCAGAATGCACAGGTACACTTGTTT  AGAGGTCTAGATTTAGTTGATGTGAGAGTTGTGAGATTCGTG | PCR amplification  for In-Fusion  clone |
| InFusR | AAACGACAATCTGATCATGAGCGGAGAATTAAGGGAGTCACGTT  ATGACCTCTAGACCTCTAAACAAGTGTACCTGTGCATTCTGGG |  |
| HisproF1^b^ | CACACACAAACCTCTCTCCCACT | T-DNA insert detection |
| TrpCR1^b^ | CAAATGAACGTATCTTATCGAGATCC |  |
| HisproF2^b^ | GTGTTCACTCGCATCCCGC | T-DNA insert detection |
| TrpCR2^b^ | AGGCACTCTTTGCTGCTTGG |  |
| **Primers used for RT-qPCR** | |  |
| GKRTF | GTCTCCCCATTACAACCTACTTC | GK RT-qPCR |
| GKRTR | CACCGTCCTTTCCTCCTGT |  |
| G3PD1RTF | ATTTGGCCGGAGAGCCG | G3PD1 RT-qPCR |
| G3PD1RTR | AGGAATGGGGAGGCAGCTC |  |
| G3PD2RTF | AATCATCGGATCAGGCAACT | G3PD2 RT-qPCR |
| G3PD3RTR | TGCTCCTCAAAGACCCACAT |  |
| G3PD3RTF | GAGACCCAACAAGACCTCACG | G3PD3 RT-qPCR |
| G3PD3RTR | TGGCGTCGGCAGATTCAG |  |
| G6PDRTF | GCGTACAAAGATGGATCGG | G6PD RT-qPCR |
| G6PDRTR | TGAAAGCCGTCGTCTGTG |  |
| PGDRTF | ACAATGGCTACACCGTCTGC | PGD RT-qPCR |
| PGDRTR | GACCTTACGAGGGCGCTTC |  |
| ICDHRTF | TTGCCAACAAAATCAACGGAG | ICDH RT-qPCR |
| ICDHRTR | GATCGACATAGGGGAGAATGAGC |  |
| 18SRTF^c^ | CGTACTACCGATTGAATGGCTTAG | Internal control for  RT-qPCR |
| 18SRTR^c^ | CCTACGGAAACCTTGTTACGACT |  |

^a^ Underlined sequences indicate the additional restriction sites.

^b^ As described by Hao et al. ([Hao et al., 2014b](#_ENREF_18)).

^c^ As described by Hao et al. ([Hao et al., 2014a](#_ENREF_17))


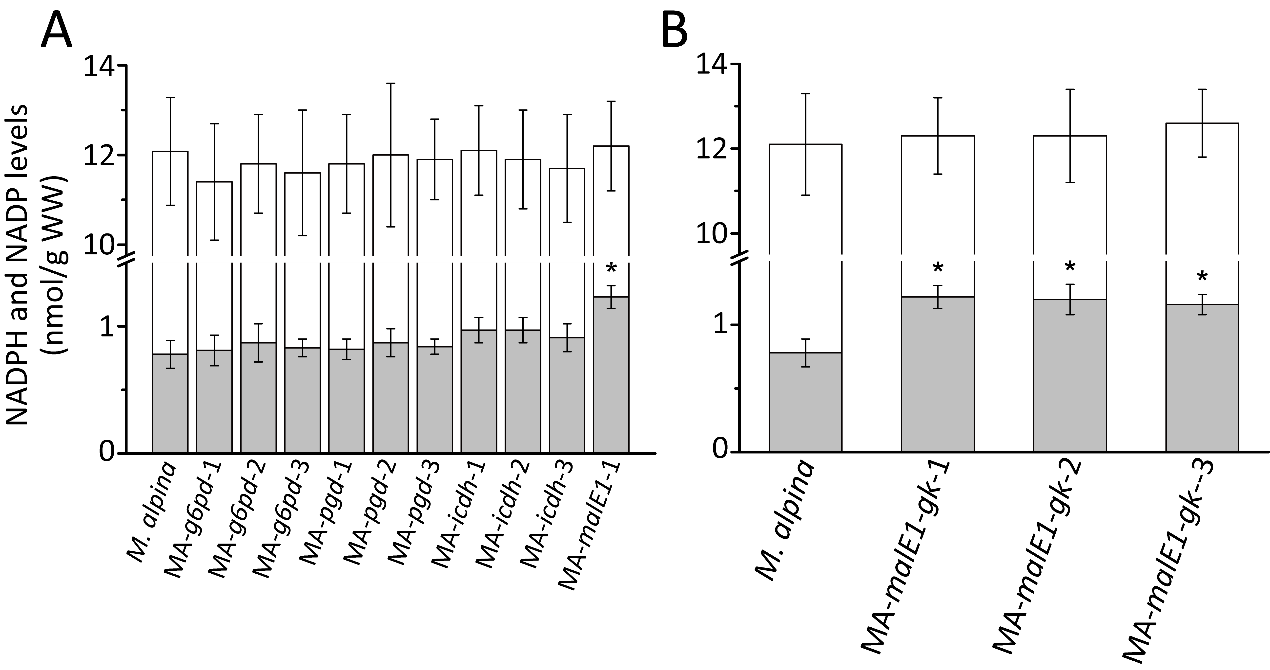


**Fig. S1.** NADPH (gray bars) and NADP (white bars) levels of different *M. alpina* strains. Strains were cultured in 500 mL shaking flasks containing 200 mL Kendrick medium supplemented with 50 g/L glycerol for 168 h at 200 rpm. Three independent experiments were performed, and the error bars represent standard deviations. ^🞲^ *p* < 0.05 compared to wild type.
